# An Endangered Arboreal Specialist, the Western Ringtail Possum (*Pseudocheirus occidentalis*), Shows a Greater Genetic Divergence across a Narrow Artificial Waterway than a Major Road

**DOI:** 10.1371/journal.pone.0146167

**Published:** 2016-01-19

**Authors:** Kaori Yokochi, Winn Jason Kennington, Roberta Bencini

**Affiliations:** School of Animal Biology, The University of Western Australia, Crawley, Western Australia, Australia; University of York, UNITED KINGDOM

## Abstract

The fragmentation of habitats by roads and other artificial linear structures can have a profound effect on the movement of arboreal species due to their strong fidelity to canopies. Here, we used 12 microsatellite DNA loci to investigate the fine-scale spatial genetic structure and the effects of a major road and a narrow artificial waterway on a population of the endangered western ringtail possum (*Pseudocheirus occidentalis*) in Busselton, Western Australia. Using spatial autocorrelation analysis, we found positive genetic structure in continuous habitat over distances up to 600 m. These patterns are consistent with the sedentary nature of *P*. *occidentalis* and highlight their vulnerability to the effects of habitat fragmentation. Pairwise relatedness values and Bayesian cluster analysis also revealed significant genetic divergences across an artificial waterway, suggesting that it was a barrier to gene flow. By contrast, no genetic divergences were detected across the major road. While studies often focus on roads when assessing the effects of artificial linear structures on wildlife, this study provides an example of an often overlooked artificial linear structure other than a road that has a significant impact on wildlife dispersal leading to genetic subdivision.

## Introduction

Roads and other artificial linear structures, such as railways, powerline corridors, and artificial waterways are thought to inhibit movements of some animals, leading to the fragmentation of populations, increased inbreeding, and loss of genetic diversity [1]. In a review on the genetic effects of roads on wildlife populations, Holderegger and Di Giulio [2] found that fragmentation of habitats by roads quickly decreased genetic diversity within populations and increased genetic divergence between populations in a wide range of species including invertebrates, amphibians and mammals. Clark et al. [3] and Epps et al. [4] also found that relatively recently built roads limited the dispersal and increased genetic divergence of timber rattle snakes (*Crotalus horridus*) and bighorn sheep (*Ovis canadensis nelsoni*). Since inbreeding and reductions in genetic diversity increase the risk of extinction of isolated populations [5], it is crucial to consider the impacts of artificial linear structures when developing management strategies for threatened species.

Strictly arboreal species are thought to be more vulnerable than the majority of terrestrial species to the impacts of artificial linear structures without canopy connections because many of them tend to avoid descending to the ground [6, 7]. The western ringtail possum (*Pseudocheirus occidentalis* Thomas 1888) is a medium-sized nocturnal marsupial endemic to the southwest of Western Australia, the only biodiversity hotspot on mainland Australia [8]. This species is likely to be susceptible to the negative impacts of artificial linear structures due to their known sedentary nature and strong fidelity to canopies [9, 10]. Studies on their movements suggest that their dispersal range is small [9]. They also have small home ranges (< 0.5 ha), and a road and an artificial waterway have been found to restrict their movements [10].

Over the last few decades *P*. *occidentalis* has gone through a dramatic decline in numbers and range due to anthropogenic factors such as habitat destruction and fragmentation and the impact of introduced predators [11, 12]. The Bunbury–Busselton region in the southwest of Western Australia is one of the last strongholds for this species. However, it is one of the fastest growing regions in Australia [13], and suitable habitat for the possums is disappearing due to the rapid urbanisation. Despite its endangered conservation status, relatively little is known about the population structure of *P*. *occidentalis* [12]. The only genetic studies done to date are a phylogenetic study using mitochondrial DNA, which supported their status as a single species, and a broad-scale population genetic study that identified three distinct populations within the current range of the species based on microsatellite markers [14].

In this study, we used microsatellite markers to investigate whether the previously reported small home ranges and limited dispersal in *P*. *occidentalis* are supported by the presence of fine-scale genetic structure. We also investigated whether a road and artificial waterway without canopy connection were associated with genetic divergences. Given the limited movements across artificial linear structures, a strong reluctance to traverse on the ground, and lack of evidence that the species voluntarily swims [10], we predicted that there would be genetic differentiation across both the road and artificial waterway.

## Materials and Methods

### Study site

**Fig 1:**
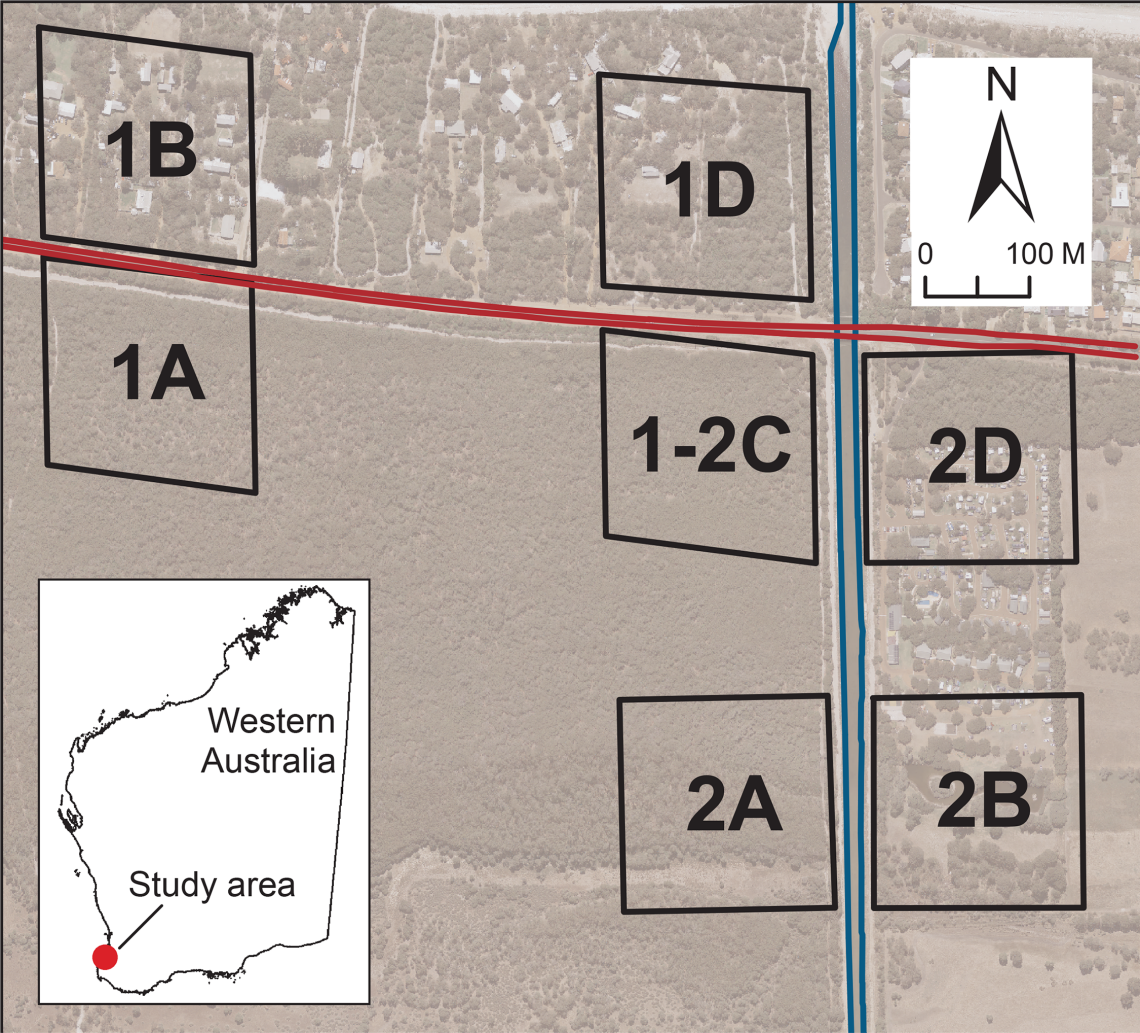
A map of a study area near Busselton, Western Australia. Red lines represent the borders of Caves Road (west to east) and blue lines represent the borders of an artificial waterway (north to south). 1A, 1B, 1-2C, 1D, 2A, 2B and 2D are 200 m × 200 m study blocks where samples from *Pseudocheirus occidentalis* were collected. 1A, 1-2C and 2A were inside Locke Nature Reserve, and 1B, 1D, 2B and 2D were within partially cleared campsites. The aerial photograph of the study area is used with permission from Western Australian Land Information Authority (Midland, WA Australia).

This study was conducted in Locke Nature Reserve and surrounding campsites, 9 km west of Busselton, Western Australia (33° 39' 32'' S; 115° 14' 26'' E), where the habitat dominated by peppermint trees (*Agonis flexuosa*) is supporting a high density of *P*. *occidentalis* [15, 16]. We set up seven 200 m x 200 m study blocks, 1A, 1B, 1-2C, 1D, 2A, 2B and 2D, chosen so that they were small enough to fall within boundaries of campsites, large enough to contain a sufficient number of individuals for sampling, and far enough from each other within continuous habitat to prevent individuals from including multiple blocks in their home ranges (Fig 1). Caves Road, running from east to west, separated the nature reserve in the south from campsites in the north with no canopy connection (Fig 1). A record of this road as a narrow gravel road exists as early as the 1930s, and it became a sealed 15 m wide single carriageway approximately 50 years ago in the 1960s. With the cleared verges on both sides and no branches in contact across it, this unfenced road provided a 25 m canopy gap. The recorded traffic volume on Caves Road was about 6,000 vehicles per day in 2008 [17]. However, the traffic volume on this road is highly seasonal, and it can be up to 15,000 vehicle per day during the peak holiday season in summer (G. Zoetelief, Main Roads WA, Pers. Comm.). On the eastern edge of the reserve, an artificially altered part of the Buayanyup River (“artificial waterway”) ran from north to south, separating the reserve in the west from a campsite in the east (Fig 1). This 30 m wide waterway was built approximately 80 years ago in the 1930s to prevent flooding in the area, and contained water all year around. With cleared paths on both banks and no branches overhanging, this artificial waterway provided a permanent 45 m gap in the canopy.

### Sample collection and microsatellite genotyping

Between March 2010 and November 2012, we captured a total of 145 adult possums by using a specially modified tranquiliser dart gun with darts containing a dose of 11–12 mg/kg of Zoletil 100^®^ (Virbac Australia, Milperra, NSW Australia). Sample sizes within each block ranged from 10 to 32 (1-2C: *n* = 12, 1A: *n* = 26, 1B: *n* = 32, 1D: *n* = 10, 2A: *n* = 31, 2B: *n* = 22, 2D: *n* = 12). Sample collection followed the methods developed by P. de Tores and reported by Clarke [18]. A thin slice of ear tissue was removed from each animal under anaesthesia using Isoflurane, and each tissue was stored in either dimethyl sulfoxide solution [19] or 90% ethanol until DNA was extracted. Coordinates of each capture location were recorded using a handheld GPS unit (Mobile Mapper Pro®, Magellan Navigation, Inc. California USA). All procedures for capturing and handling animals followed the Australian code of practice for the care and use of animals for scientific purposes [20], and were approved by the Animal Ethics Committee at The University of Western Australia (RA/3/100/539 and RA/3/100/1213). Permits to access Locke Nature Reserve (CE003434) and to capture the possums for scientific purposes (SF008419) were obtained from Western Australian Department of Parks and Wildlife.

We extracted DNA from each sample using Qiagen DNeasy Blood and Tissue kit (Qiagen, Venlo, Netherlands) following the manufacturer’s instructions. Concentration and quality of each DNA sample were then determined using a NanoDrop ND-1000 spectrophotometer (Thermo Fisher Scientific Inc., Massachusetts, USA). We used 12 species-specific microsatellite markers (A1, A106, A119, A122, A127, A2, A6, B104, C111, D104, D113, and D114) following PCR conditions described by Wilson et al. [21]. Genotypes at each locus were determined using an ABI 3700 Genetic Analyzer with a GeneScan-500 LIZ dye size standard (Applied Biosystems Inc., California, USA).

### Data analysis

The presence of null alleles was assessed for each locus using Microchecker v.2.2.3 [22]. Genetic diversity within each block was quantified by calculating allelic richness (*A*_*R*_) and Nei [23]’s estimator of gene diversity (*H*) within Fstat v.2.9.3 software package [24]. Deviations from random mating were assessed using randomization tests, with results characterized with the inbreeding coefficient (*F*_*IS*_) statistic. Significantly positive *F*_*IS*_ values indicate a deficit of heterozygotes relative to a random mating model, while negative results indicate an excess of heterozygotes. Genotypic disequilibrium was tested between each pair of loci within each study block. *F*_*IS*_ values and tests for deficits in heterozygotes and genotypic equilibrium were calculated using the Fstat v.2.9.3. Tests for differences in genetic diversity and *F*_*IS*_ among study blocks were performed using Friedman’s ANOVA and Wilcoxon rank tests with the R v.3.0.1 statistical package [25].

We performed a Spatial Autocorrelation (SA) analysis on the samples collected from the nature reserve only (blocks 1A, 1-2C and 2A, *n* = 69) to investigate how the genetic similarity of individuals changed over geographical distance within continuous vegetation (i.e. whether a fine-scale genetic structure was present without the presence of artificial linear structures). The result from this analysis would also tell us whether distances that were the same as the widths of the road and artificial waterway were large enough to cause genetic divergence in continuous vegetation. We performed SA analyses using GenAlEx v.6.501 [26] with the results presented in two different ways. Firstly, mean genetic correlation coefficients (*r*) were calculated and plotted over a range of distance classes increasing at 100 m intervals to obtain autocorrelograms. Secondly, because estimates of *r* are influenced by the size of distance classes [27], we also performed Multiple Distance Class (MDC) analyses to calculate and plot *r* for a series of increasing distance class sizes. Decreasing *r* with increasing distance interval class indicates significant positive spatial structure, and the distance interval class at which *r* is no longer greater than zero represents the limit of detectable genetic structure [27]. Tests for statistical significance were carried out by random permutation and calculating the bootstrap 95% confidence limits of *r* using 1000 replicates. We also conducted 2-Dimensional Local Spatial Autocorrelation (2DLSA) analysis using GenAlEx. This analysis investigates uniformity of spatial autocorrelation over the study area by estimating local autocorrelation (*lr*) by comparing each individual with its nearest neighbours [28]. Calculations of *lr* were made using the nearest five individuals with statistical significance determined using permutation tests.

Population structure across the whole study area was assessed using pairwise *F*_ST_ values and Bayesian clustering analysis. Pairwise *F*_ST_ values and tests for genetic differentiation between blocks were calculated using Fstat. We performed Bayesian clustering analysis using Structure v.2.3.4 [29]. This method identifies genetically distinct clusters (*K*) based on allele frequencies across all loci. Analyses were based on an ancestry model, which assumes admixture and correlated allele frequencies with study blocks used as prior information about the origin of the samples. A burn-in period of 100,000 and 1,000,000 Markov Chain Monte Carlo (MCMC) iterations were used for 10 replicate runs for each number of clusters (*K*) ranging from 1 to 10. We then determined the most likely value of *K* using the Δ*K* method of Evanno et al. [30] implemented in Structure Harvester v.0.6.93 [31]. Each individual’s average membership to *K* clusters from 10 replicate runs was calculated and re-organised using Clumpp v.1.1.2 [32] and visualised using Distruct v.1.1 [33].

The effects of artificial linear structures were then examined by comparing mean relatedness values between pairs of individuals on the same side of the road or waterway with relatedness values between pairs of individuals on opposite sides of the road or waterway. The effect of the road was examined without data from individuals on the other side of the waterway (2B and 2D) to remove the effect of the waterway, and the effect of the waterway was examined without data from those on the other side of the road (1B and 1D) to remove the effect of the road. We calculated pairwise relatedness values using the method of Queller and Goodnight [34] with GenAlEx and tested for differences between classes (within blocks, same or opposite sides of linear structure) by comparing 95% confidence limits (CLs). The CLs were calculated by resampling values within each class (with replacement) and recalculating the mean 1000 times. We also performed a permutation test to determine whether mean pairwise relatedness values for each class were significantly different to zero. This was done by randomly shuffling pairwise relatedness values across classes and recalculating the mean 1000 times. We then compared the observed value to the randomised distribution of mean values to calculate the probability of the result.

## Results

### Genetic variation within blocks

Two loci were identified as having null alleles (locus A119 in block 1B and locus A1 in blocks 2A and 2D). Since there was no consistent pattern in the presence of null alleles (i.e. the loci with null alleles were not the same across study blocks), all loci were retained for further analysis. No *F*_*IS*_ values were significantly different from zero, indicating that the groups of possum in all blocks were in Hardy-Weinberg Equilibrium. There was no evidence of genotypic disequilibrium between any pair of loci within any study block based on 9240 permutations after correcting for multiple comparisons by using an adjusted *P* value of 0.000108. There were also no significant differences in allelic richness (χ^2^ = 8.73, *P* = 0.189, *d*.*f*. = 6), gene diversity (χ^2^ = 8.55, *P* = 0.200, *d*.*f*. = 6) or *F*_*IS*_ (χ^2^ = 4.12, *P* = 0.660, *d*.*f*. = 6) among study blocks (Table 1).

**Table 1:** Genetic variation among *Pseudocheirus occidentalis* within each study block.

Block	*N*	*A*_*R*_	*H*	*F*_*IS*_
1-2C	12	3.5 (0.3)	0.62 (0.03)	–0.02
1A	26	3.4 (0.3)	0.57 (0.04)	–0.02
1B	32	3.7 (0.3)	0.60 (0.03)	0.01
1D	10	3.3 (0.3)	0.56 (0.05)	0.00
2A	31	3.7 (0.3)	0.61 (0.04)	0.04
2B	22	3.6 (0.3)	0.60 (0.03)	–0.01
2D	12	3.9 (0.3)	0.64 (0.03)	0.06

Standard errors are in parentheses. *N* is the mean sample size per locus, *A*_*R*_ is the mean allelic richness based on a sample size of 10 individuals, *H* is the mean gene diversity, and *F*_*IS*_ is the inbreeding coefficient. No *F*_*IS*_ values were significantly different from zero.

### Fine-scale genetic structure within continuous habitat

**Fig 2:**
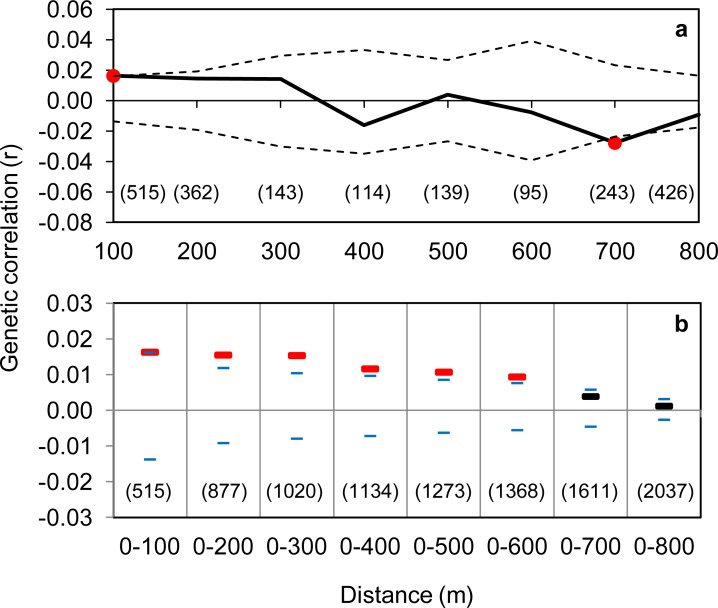
(a) A correlogram plot and (b) a multiple distance class plot based on 69 *Pseudocheirus occidentalis* in continuous habitat in Locke Nature Reserve near Busselton, Western Australia. Dotted lines (a) and small blue markers (b) represent upper and lower 95% confidence intervals around zero. Red circle markers on the solid line (a) and red markers (b) are the genetic correlation values (*r*) that differ significantly from zero based on bootstrap resampling. Sample sizes are shown in parentheses.

**Fig 3:**
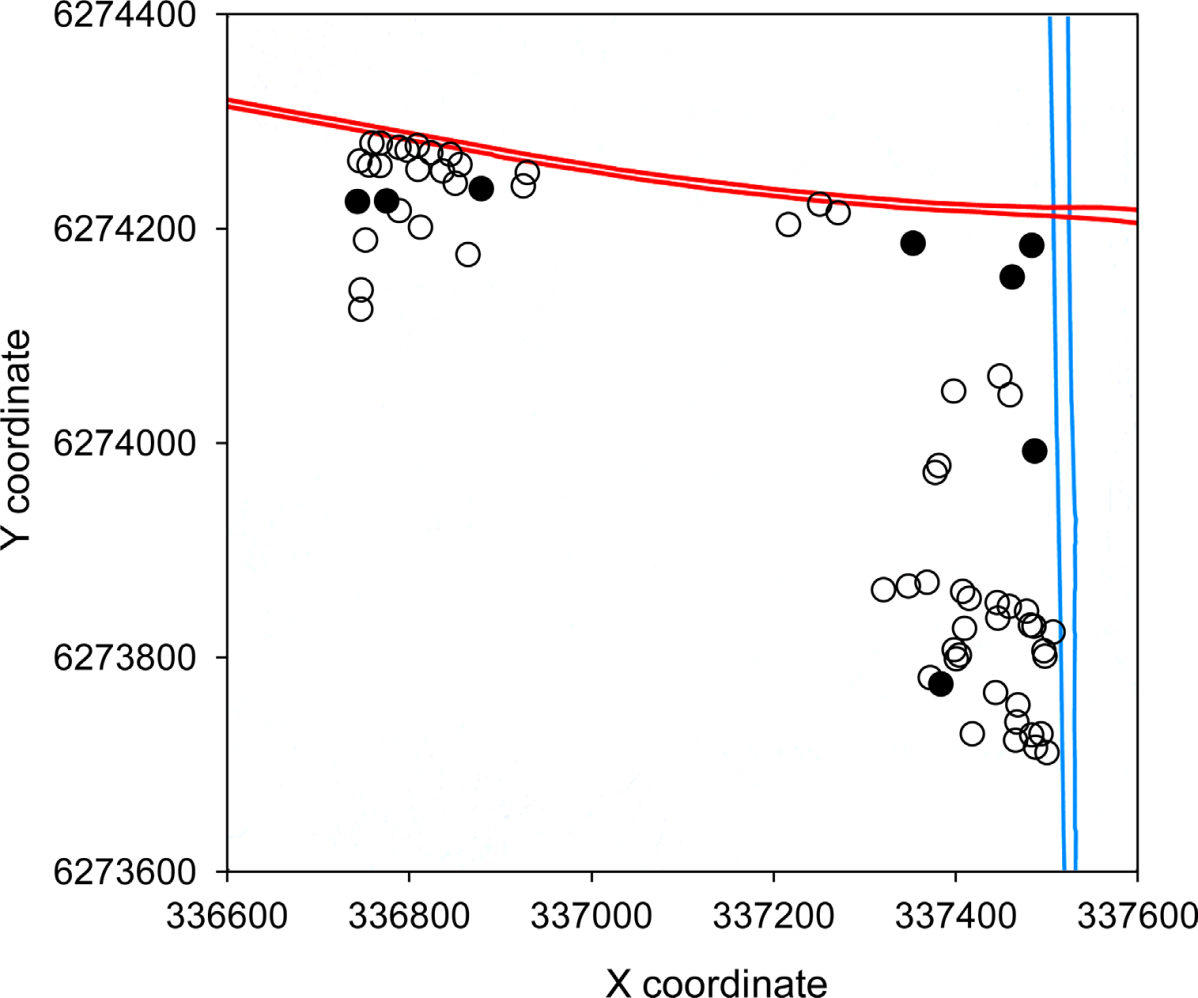
Plot of two-dimensional local spatial autocorrelation analyses of *Pseudocheirus occidentalis* sampled in Locke Nature Reserve near Busselton, Western Australia. Red lines represent the borders of Caves Road (west to east) and blue lines represent the borders of an artificial waterway (north to south). The area south of Caves Road and west of the waterway is Locke Nature Reserve. Markers represent geographical locations of the local spatial autocorrelation analyses with significantly positive (solid symbols) or non-significant values (open symbols) based on five nearest neighbours. Coordinates are based on GDA 94 projection (zone 50).

Fine-scale spatial genetic structure was detected within continuous habitat. In SA analysis, the genetic correlation coefficient (*r*) was significantly positive up to 100 m, and it intercepted zero at 347 m (Fig 2A). The MDC analysis showed significantly positive *r* values over distances up to 600 m (Fig 2B). All size class bins were well represented, with a minimum of 95 and 515 pairwise comparisons per bin for the SA analyses and MDC analysis respectively. The 2DLSA analysis revealed clusters of positive genetic correlation in all three blocks, indicating that positive genetic structure was not confined to one particular area within the nature reserve (Fig 3).

### Population structure across the study area and the effect of artificial linear structures

Significant genetic divergences were observed between 14 (67%) pairs of blocks. These pairs of blocks occurred on the same and opposite sides of Caves Road, as well as the same and opposite sides of the artificial waterway (Table 2). Pairwise *F*_*ST*_ values ranged from 0.017 to 0.079, with the highest *F*_*ST*_ occurring between blocks 1D and 2D, which were separated by both the road and waterway (Table 2).

**Table 2:** Pairwise *F*_ST_ values (below diagonal) and *P*-values from tests of differentiation (above diagonal) between blocks.

	1-2C	1A	1B	1D	2A	2B	2D
1-2C	-	0.0010	0.0019	0.0010	0.0081	0.0005	0.0095
1A	**0.050**	-	0.0010	0.0148	0.0033	0.0005	0.0005
1B	**0.050**	**0.035**	-	0.0081	0.0033	0.0005	0.0005
1D	**0.056**	0.016	0.033	-	0.0033	0.0005	0.0005
2A	0.033	0.012	0.017	0.023	-	0.0005	0.0005
2B	**0.060**	**0.023**	**0.061**	**0.040**	**0.034**	-	0.0019
2D	0.041	**0.061**	**0.047**	**0.079**	**0.036**	**0.045**	-

Significant divergences are highlighted in bold text. The adjusted significance level for multiple comparisons is 0.0024. *P*-values were obtained after 2100 permutations.

**Fig 4:**
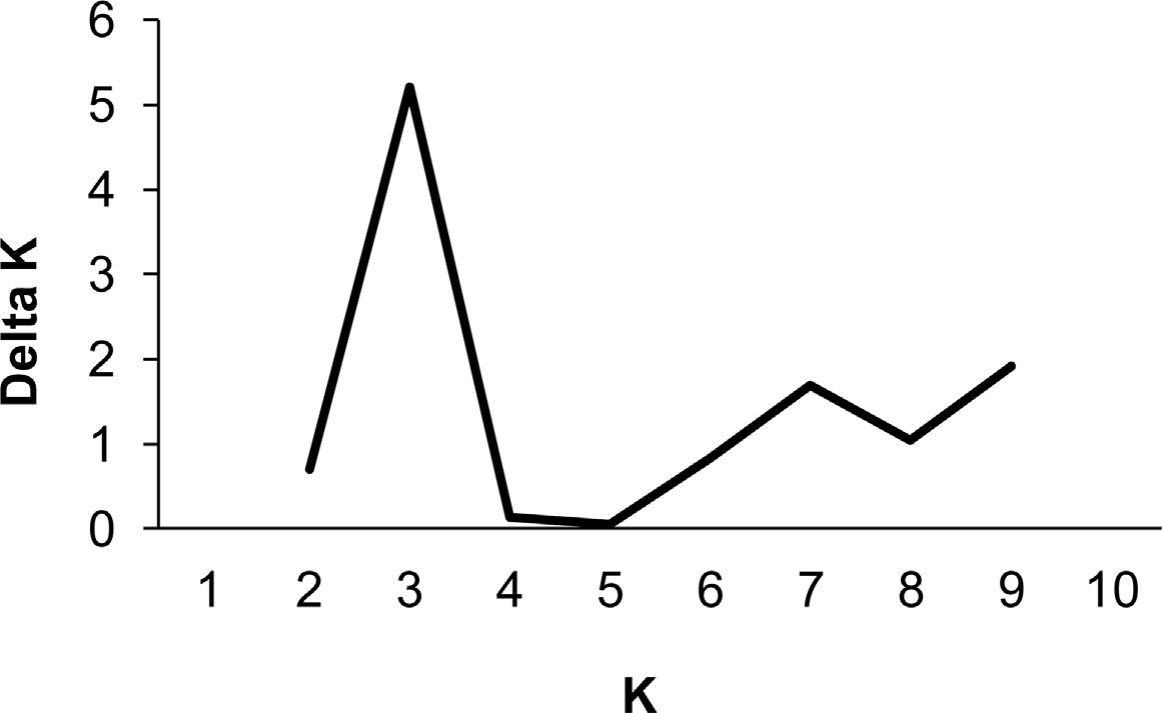
Summary of Δ*K* estimates[30] for varying numbers of genetic clusters (*K*) derived from the STRUCTURE analysis of 145 adult *Pseudocheirus occidentalis* from Busselton, Western Australia.

**Fig 5:**
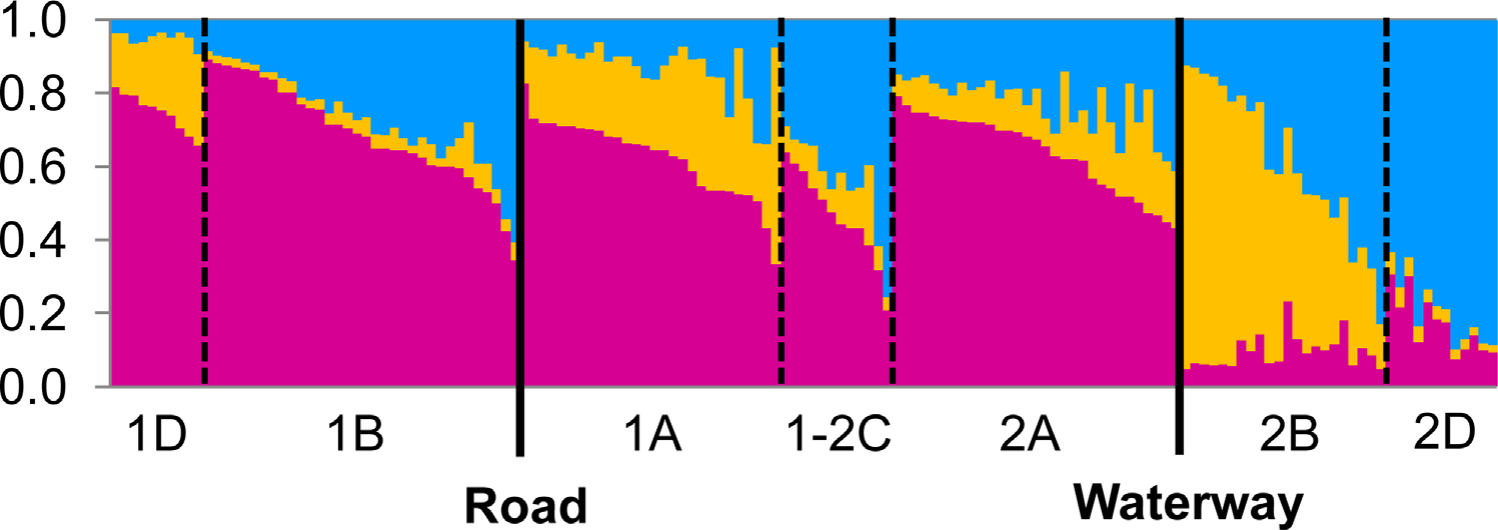
Summary of the Bayesian clustering analysis assuming three admixed populations of *Pseudocheirus occidentalis* in Busselton, Western Australia. Each column represents an individual’s estimated membership to three genetic clusters represented by different colours. Vertical dotted lines separate individuals sampled from different blocks. Vertical bold solid lines represent the presence of Caves Road and an artificial waterway.

Population structure across the whole study area was also evident with the Bayesian clustering analysis. Analysis of the Structure results using the Δ*K* method clearly identified *K* = 3 as the most likely number of genetic clusters (Fig 4). A bar plot of individuals’ memberships to each cluster showed that most individuals from the western side of the artificial waterway (blocks 1-2C, 1A, 1B, 1D and 2A) were predominantly assigned to cluster 1 (shown in pink in Fig 5), whereas those from the eastern side (blocks 2B and 2D) were predominantly assigned to clusters 2 (yellow) and 3 (blue) respectively (Fig 5).

**Fig 6:**
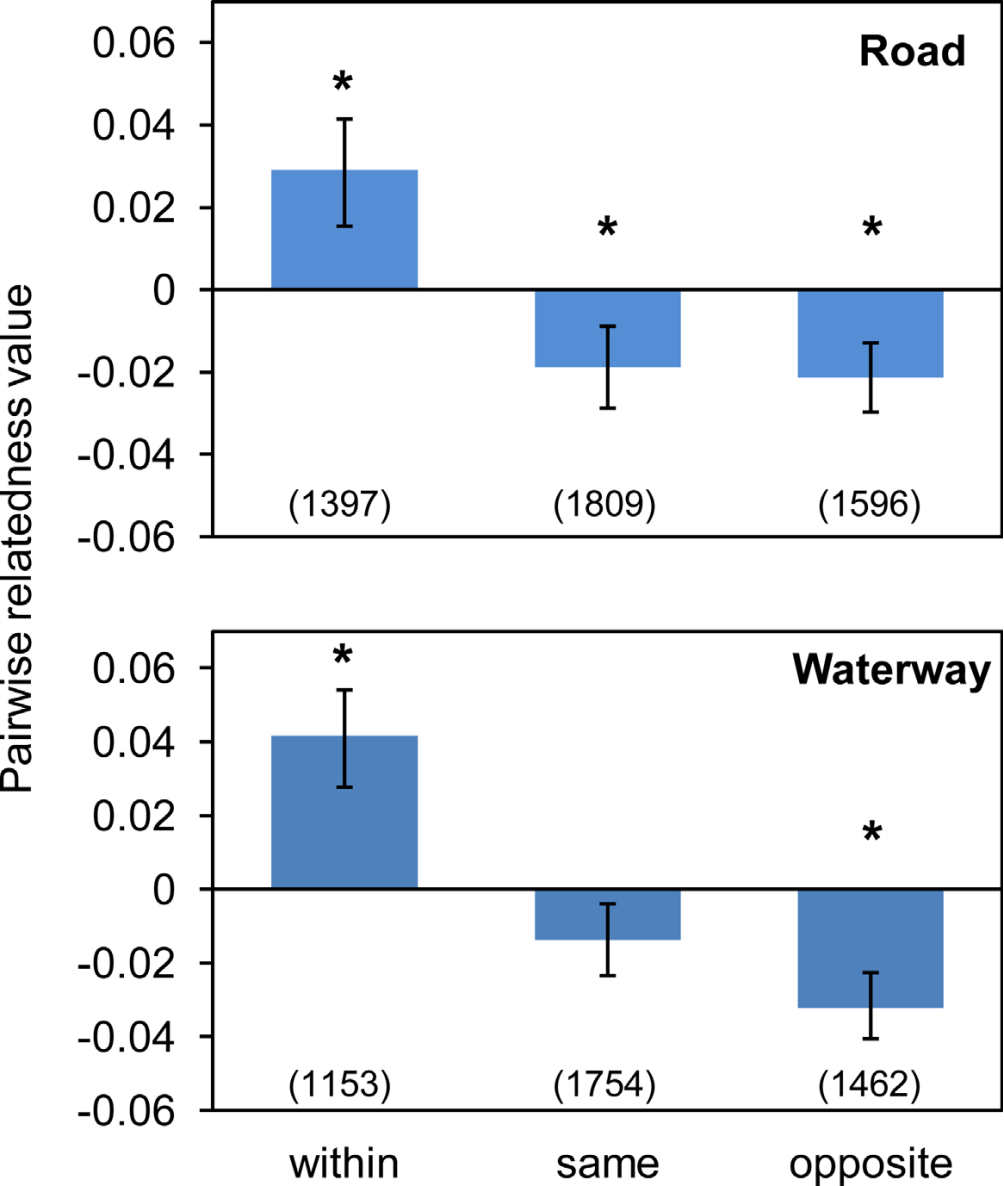
Mean pairwise relatedness values [34] for *Pseudocheirus occidentalis* individuals sampled from the same block (“Within”), different blocks on the same side of an artificial barrier (“Same”) and opposite sides of an artificial barrier (“Opposite”). Error bars represent the 95% confidence levels determined by bootstrap resampling, and values in parentheses are the numbers of pairwise comparisons from which the average pairwise relatedness values were calculated. Asterisks represent mean values significantly different to zero determined by a permutation test.

The pairwise relatedness analysis also indicated that the individuals separated by the artificial waterway were significantly less related to each other than the individuals on the same side of the waterway (Fig 6). This was evident from the 95% CLs for the same side of the waterway not overlapping the mean value for opposite sides of the waterway and vice-versa. By contrast, there were no differences between mean values for the same and opposite sides of the road. To test whether the greater distances between individuals from the opposite sides of the waterway were contributing to lower pairwise relatedness values, we recalculated the mean for this class without the furthest block from the waterway (i.e. block 1A). We found there was no difference to the previous result–CLs did not overlap with means–indicating the greater separation between individuals did not explain the significantly lower pairwise relatedness values between individuals from different sides of the waterway. The permutation tests revealed that all mean values were significantly different to zero with the exception of the class representing the same side of the waterway (Fig 6).

## Discussion

The fine-scale population structure detected in this study confirms that dispersal in *P*. *occidentalis* is limited. The maximum distance at which a significantly positive genetic structure was detected in continuous habitat with SA and MDC analyses was 600 m. This finding is consistent with the sedentary nature of this species observed in previous telemetry studies [9, 10, 18]. Fine-scale population structure was also evident with the pairwise relatedness values, which were significantly higher between individuals from the same blocks than between individuals from different blocks. Furthermore, the fine-scale genetic structuring was observed equally in all study blocks within the nature reserve, indicating that this genetic structuring was not restricted to one part of the nature reserve. Similar patterns of positive fine-scale genetic structuring have been reported in other small to medium sized mammals, such as the Australian bush rat (*Rattus fuscipes* [27]), the brush-tailed rock-wallaby (*Petrogale penicillata* [35]), the Eurasian badger (*Meles meles* [36]), the southern hairy-nosed wombat (*Lasiorhinus latifrons* [37]), and the squirrel glider (*Petaurus norfolcensis*, [38]). All of these species are thought to have short dispersal distances due to their philopatry or small body size, both of which may have contributed to the positive genetic structuring found in this *P*. *occidentalis* population.

SA analysis on another species of possum, the common brushtail possum (*Trichosurus vulpecula*) found positive spatial structure up to distances of 896 m and 454 m for males and females, respectively [39]. Male-biased dispersal of up to 10 km was also recorded for this species in field studies [40]. Another arboreal marsupial, the koala (*Phascolarctos cinereus*) has also been recorded to disperse up to 10.6 km [41] with a spatial autocorrelation analysis showing no evidence of limited dispersal [42]. In this study, we could not investigate the fine-scale genetic structure of males and females separately due to small sample sizes. Such analyses are required to test the hypothesis that dispersal in *P*. *occidentalis* is driven by males, which is based on observations of only a few individuals [9]. Nevertheless, the presence of fine-scale population structure at short geographical distances (300–400 m) highlights the exceptionally high level of philopatry in *P*. *occidentalis* even compared with other arboreal marsupials, highlighting their high susceptibility to the impacts of habitat fragmentation.

In support of our prediction about the effect of the artificial waterway, both the pairwise relatedness and Bayesian cluster analyses indicated that possums separated by the artificial waterway were genetically distinct, suggesting its capability to restrict gene flow among the possums. The waterway was much narrower than the spatial scale at which positive genetic structure was detected within continuous vegetation, and this rules out the possibility that the genetic divergence across the waterway was purely due to geographical distance. Thus, it appears that even a 30 m wide artificial waterway is sufficient to restrict gene flow in *P*. *occidentalis*, enhancing population structure over short distances.

On the other hand, the road did not seem to have an apparent effect on the genetic connectivity of *P*. *occidentalis*, according to the results from pairwise relatedness and Bayesian cluster analyses, even though it was found to be restricting the movements of possums in a previous study [10]. Caves Road is a busy road without canopy connection. However, it is not fenced and possum roadkills have been recorded in the area [43], indicating that some possums try to cross it. One to ten migrants per generation are considered to be enough to maintain genetic homogeneity [44], and it is possible that the small number of possums that successfully crossed the road had been sufficient to maintain enough gene flow to prevent genetic divergence. The gap in the vegetation across the road was 20 m narrower than that across the artificial waterway, which may also have contributed to the apparently higher permeability of the road. Moreover, Caves Road was bituminised only 40 to 50 years ago, with significant increases in traffic volumes occurring only recently, so its barrier effect may not have had sufficient time to result in genetic divergences detectable with the microsatellite loci used in this study. What we detect in the genetic structure of animals today reflects historic rather than present events, and depending on the circumstances, it could take tens and hundreds of generations for the effects of habitat fragmentation to become detectable [45]. Therefore, we need to be cautious when interpreting this result and still should not disregard the risk of this road causing genetic divergence within this important population of *P*. *occidentalis*.

Another possible explanation for the difference in the genetic effect of the road and waterway is the difference in effective population size of possums along these linear structures. A population with a smaller effective population size suffers a greater effect of genetic drift, which results in a greater rate of genetic divergence [46]. While this remains a possibility, a previous study has shown that the density of *P*. *occidentalis* within Locke Nature Reserve does not differ near Caves Road or the artificial waterway [47]. Our data also indicate that the levels of genetic diversity and inbreeding were not different among blocks. Therefore, it is unlikely that the difference in the level of genetic divergence between these barriers was caused by the effective population size alone.

In a study investigating genetic divergence among red-backed salamanders (*Plethodon cinereus*) occurring on different sides of roads and streams, Marsh et al. [46, 48] found that 2–7 m wide streams caused small, but significant genetic divergence. Amongst the roads, only one 104 m wide highway caused apparent genetic divergence out of six roads examined. The other five roads (13–47 m) were at least 30 years older than the highway, but much narrower and not as busy. Quéméré et al. [49] also found that the primary causes of genetic structuring in the golden-crowned sifaka (*Propithecus tattersalli*) were a river and geographical distances rather than a road. On the other hand, Estes-Zumpf et al. [50] found that none of the roads or creeks they examined caused genetic divergence in the pygmy rabbit (*Brachylagus idahoensis*) possibly due to the greater mobility of this species. These results, together with our own, indicate that different types of linear barriers can have different levels of permeability to the movement of different species, and the permeability of barriers also depends on the mobility of the species. Therefore, it is important to assess the negative impacts of a wider range of artificial linear structures especially in areas where endangered sedentary species occur.

*F*_*ST*_ values also suggested that the groups of possums on the east of the waterway were genetically different from those on the west. The samples from block 2B were significantly different from the all other blocks, probably due to the presence of the waterway and the mostly cleared land between blocks 2B and 2D. Block 2D was significantly different from all other blocks except 1-2C. There is a road bridge across the waterway north of these two blocks (see Fig 1), and it is possible that some *P*. *occidentalis* have used this bridge to cross the waterway in the past, resulting in the non-significant pairwise *F*_*ST*_ between blocks 2D and 1-2C. However, it should also be noted that the sample sizes within these blocks (*n* = 12 for both) were considerably smaller than the others, lowering the reliability of the *F*_*ST*_ value. The pairwise *F*_*ST*_ values showed inconclusive results across the road. For example, *F*_*ST*_ values between blocks 1A and 1B and blocks 1-2C and 1D were found to be significant; however, *F*_*ST*_ values between blocks 1B and 2A and blocks 1D and 2A were non-significant despite the presence of a road and greater distances between these blocks. This inconsistency may have been caused by the small sample sizes in some of the study blocks and/or the fine scale of the genetic divergence examined in this study. Individual-level analyses, such as pairwise relatedness analyses and Bayesian cluster analyses, have been suggested to be more appropriate than population-level analyses, such as estimated pairwise *F*_*ST*_, for studies examining fine-scale genetic divergence because combining individuals within populations can lead to loss of fine-scale information [51]. Given the small geographical scale of this study, the clear results shown by both of individual-level analyses are likely to be more robust than the inconsistent results shown by a population-level analysis.

All blocks had high levels of genetic variation and did not deviate from Hardy-Weinberg equilibrium, suggesting that the possums in these blocks are not experiencing a high level of inbreeding despite the presence of two barriers. This is probably because blocks were still connected with other patches of habitat outside our study area and they were not completely isolated. The different cluster membership in the Bayesian cluster analysis for blocks 2B and 2D may also be explained by these connections to other patches. Blocks 2B and 2D were isolated by the waterway from the other blocks, and were separated from each other by a mostly cleared campsite. Given the exceptionally highly sedentary and arboreal nature of this species [10], the limited canopy connection between these two blocks (Fig 1) may have been enough to cause this differentiation between neighbouring blocks. However, 2D had continuous canopy covers to the habitat east of the study area via a road reserve (Fig 1), possibly altering the allele frequencies in this area compared to those on the western side of the waterway. Block 2B was the most isolated among all blocks because the area to the East and South of this block was mostly cleared farmland (Fig 1), and this isolation seems to be reflected in its memberships to mostly yellow and blue genetic clusters (Fig 5) rather than mostly red and blue like the other blocks. To confirm this, DNA samples from possums in the area east and south of the campsite would need to be analysed; however, this was outside of the scope of our study.

Caves Road has not caused a detectable genetic divergence among possums yet, but it is restricting their movements and causing mortality [10], which also reduces genetic diversity [52]. The number and range of *P*. *occidentalis* is expected to decline dramatically in the future due to the current increasing pressure of habitat clearing and impacts of climate change [53]. Even though possums in our study area did not seem to suffer from a low genetic diversity or a high level of inbreeding at the time of sample collection, the barrier effects of the waterway and road still need to be mitigated to maximise their ability to disperse and maintain the high level of genetic diversity.

## Conclusion

Both telemetry data from a previous study [10] and genetic data from this study show that *P*. *occidentalis* have limited dispersal, making them highly susceptible to the impacts of habitat fragmentation. Indeed, we found evidence to suggest that an artificial waterway has been limiting the gene flow of *P*. *occidentalis*, resulting in genetic divergence within spatial scales of hundreds of metres. By contrast, a busy road does not seem to have had a detectable impact on population structure, though future impacts cannot be ruled out. While studies investigating negative effects of artificial linear structures on population structure tend to focus on roads, our study provides an example where an artificial waterway may be posing significantly greater genetic impacts on an endangered arboreal species than a major road. We therefore urge for more research to be conducted on the impacts of artificial linear structures other than roads, and their impacts be mitigated where significant.
